# The pupillary light reflex distinguishes between circadian and non-circadian delayed sleep phase disorder (DSPD) phenotypes in young adults

**DOI:** 10.1371/journal.pone.0204621

**Published:** 2018-09-27

**Authors:** Elise M. McGlashan, Angus C. Burns, Jade M. Murray, Tracey L. Sletten, Michelle Magee, Shantha M. W. Rajaratnam, Sean W. Cain

**Affiliations:** Monash Institute of Cognitive and Clinical Neurosciences and School of Psychological Sciences, Monash University, Melbourne, Australia; University of Texas Southwestern Medical Center, UNITED STATES

## Abstract

This study investigated the utility of the pupillary light reflex as a method of differentiating DSPD patients with delayed melatonin timing relative to desired/required sleep time (circadian type) and those with non-delayed melatonin timing (non-circadian type). All participants were young adults, with a total of 14 circadian DSPD patients (*M* = 28.14, *SD =* 5.26), 12 non-circadian DSPD patients (*M =* 29.42, *SD =* 11.51) and 51 healthy controls (*M* = 21.47 *SD* = 3.16) completing the protocol. All participants were free of central nervous system acting medications and abstained from caffeine and alcohol on the day of the assessment. Two pupillary light reflex measurements were completed by each participant, one with a 1s dim (~10 lux) light exposure, and one with a 1s bright (~1500 lux) light exposure. Circadian DSPD patients showed a significantly faster pupillary light reflex than both non-circadian DSPD patients and healthy controls. Non-circadian patients and healthy controls did not differ significantly. Receiver operating characteristic curves were generated to determine the utility of mean and maximum constriction velocity in differentiating the two DSPD phenotypes, and these demonstrated high levels of sensitivity (69.23–-100%) and specificity (66.67–91.67%) at their optimal cut offs. The strongest predictor of DSPD phenotype was the mean constriction velocity to bright light (AUC = 0.87). These results support the potential for the pupillary light reflex to clinically differentiate between DSPD patients with normal vs. delayed circadian timing relative to desired bedtime, without the need for costly and time-consuming circadian assessments.

## Introduction

Delayed sleep phase disorder (DSPD) is categorised as a circadian rhythm sleep disorder according to the International Classification of Sleep Disorders (ICSD2 [1]. However, the delayed sleep-wake behaviour seen in those with DSPD may have different physiological causes between patients. Recently, two distinct phenotypes within the DSPD population were characterised [2]: one with a circadian delay, and one with typical circadian timing relative to the desired sleep time. Thus, nearly half of patients diagnosed with this circadian rhythm sleep disorder do not have a circadian basis to their sleep problems, and are therefore potentially being misclassified.

Current recommended treatments for DSPD include interventions which are designed to normalise circadian timing, such as morning bright light therapy or melatonin administration [3]. Each of these are intended to advance circadian timing. However, the impact of these treatments differs depending on the biological time at which they are administered. Therefore, their impact will differ between circadian and non-circadian DSPD patients when given relative to sleep (as opposed to biological time), and these two distinct phenotypes would likely benefit from different treatment approaches. Clinically viable methods of differentially diagnosing these distinct phenotypes would allow for individualized treatments.

A potential mechanism for the development of abnormal circadian entrainment in DSPD patients is altered sensitivity of the circadian system to light. Environmental light cues play a critical role in determining circadian timing [4], with light exposure in the evening resulting in delays in circadian phase, and light exposure in the late night to early morning resulting in advances in circadian phase [5]. It has been demonstrated that DSPD patients exhibit an increased sensitivity to night time light exposure, whereby they experience increased melatonin suppression relative to healthy controls [6, 7]. It is hypothesised that this increased sensitivity, combined with exposure to light at night results in the development and maintenance of circadian delays in these patients. However, whether hypersensitivity to light exists in both the circadian and non-circadian phenotypes of DSPD has not been studied.

The pupillary light reflex (PLR) describes the acute response of the pupil to a pulse of light. In mammals, the pupillary light reflex is mediated by retinal cells including rods, cones and melanopsin-containing intrinsically-photosensitive retinal-ganglion cells (ipRGCs) [8]. Melanopsin-containing ipRGCs also play a critical role in circadian entrainment by transmitting light information to the suprachiasmatic nucleus (SCN) [9, 10], the master circadian clock [11, 12]. As the PLR is mediated by ipRGCs, it may reflect the sensitivity of the circadian system to light input. Recent studies have revealed relationships between pupillometric outcomes and sleep-wake parameters such as diurnal preference and sleep-wake timing [13, 14]. However, pupil responses have not been related to a clinical sleep disorder diagnosis. The current study investigated the utility of a brief PLR measurement in distinguishing DSPD phenotypes. Additionally, we examined differences in the PLR between DSPD patients and healthy controls. Given the likely association between the PLR and circadian light sensitivity, we hypothesised that the circadian DSPD patients would exhibit a significantly faster PLR than the non-circadian DSPD patients. Additionally, we hypothesised that the circadian DSPD patients would exhibit a significantly faster PLR than healthy controls, indicating a hypersensitivity to light.

## Materials and methods

Ethical approval was obtained from the Monash University Human Research Ethics Committee (MUHREC). All participants provided written informed consent and were reimbursed for their time.

### Participants

A total of 26 patients completed the study, 14 circadian DSPD patients, 12 non-circadian DSPD patients. Circadian and non-circadian DSPD patients were categorised according to our previously developed criteria [2], using a combination of physician diagnosis and circadian phase assessments. In summary, all patients initially met with a sleep physician to confirm a diagnosis of DSPD (ICSD-2 criteria). Potential participants who did not meet criteria for DSPD were excluded. All eligible patients subsequently underwent an evening in-laboratory assessment of salivary melatonin levels, to determine melatonin onset time. Patients were categorised as circadian DSPD if they exhibited a dim-light melatonin onset (DLMO) time of up to 30 minutes before, or any time after their desired or required sleep time. Patients were classified as non-circadian DSPD if they had a DLMO time of more than 30 minutes before their desired bedtime. We classified our patients based on melatonin onset relative to desired bedtime (DBT), as an inability to initiate sleep at DBT forms part of the criteria for the diagnosis of DSPD [1]. Melatonin onset occurs an average of 2 hours before bedtime in healthy individuals [15]. Therefore, it was determined that if melatonin onset occurred at least 30 minutes prior to desired bedtime, an inability to initiate sleep was not likely to be caused by a delay in the circadian drive for sleep (i.e., the patient is considered ‘non-circadian’). Alternatively, if melatonin onset occurred less than 30 minutes before DBT, the patients is likely to be experiencing sleep-onset difficulties due to this delay in circadian phase.

At the time of the PLR assessment, participants were not taking central nervous system acting medications. We recruited participants from a previously established cohort of DSPD participants [2], approximately 1.5–2 years (*M =* 1.74 years, *SD =* 0.51) after their initial diagnosis. Thus, the clinical diagnoses and circadian assessments used to classify them were retrospective. We compared PLR results from DSPD patients with those of 51 healthy volunteers from a database of healthy sleepers. Healthy participants were not taking any medications and had no diagnosed sleep disorders or psychiatric conditions. Participant demographics are presented in *Table 1.*

**Table 1:** Participant demographics.

	Age	Sex M-F	DLMO 10pMol	Habitual Bedtime	Phase Angle
Circadian DSPD	28.14 (5.26)	9–5	23:09 (72.69)	25:07 (64.05)	1.96 (1.43)
Non-circadian DSPD	29.42 (11.51)	5–7	20:52 (60)	24:10 (63.45)	3.15 (1.07)
Healthy controls	21.47 (3.16)	32–19	20:32 (68.36)	23:06 (47.10)	2.57 (0.99)
*p* value	< .001	.38	< .001	< .001	.029
Sig pairwise comparisons	HvNC** HvC**		CvNC** CvH**	CvNC** CvH** NCvH**	CvNC*

*p < .05

**p < .01, error denoted as SD (in minutes for clock times). Between group comparisons are one-way ANOVAs, post-hoc tests are reported with a Bonferroni-Holm correction. M:F proportions compared between groups using a *chi-square* statistic. C = circadian DSPD, NC = non-circadian DSPD, H = healthy controls, phase angle = difference in hours between DLMO and bedtime. For DLMO and Phase angle non-circadian *n* = 11 as DLMO could not be determined (occurred prior to the measurement period) for *n* = 1.

### Procedure

Participants were instructed to refrain from drinking alcohol or consuming any caffeine for 24 hours prior to their PLR assessment. The assessment was ~12 minutes in duration and was conducted between 2 and 8 hours after habitual wake time. This time frame was selected as ipRGC mediated pupil responses are stable during this period [16]. Participants were initially dark-adapted for 10-minutes, before two light pulses (dim followed by bright in all cases) were delivered. There was an inter-stimulus-interval of 2 minutes between the onset of light stimuli. Dark adaptation involved sitting in a completely dark, quiet room, with the screen of the device (which is viewable to only the researcher) being filtered with neutral density filters (LEE, Lightmoves, VIC, AU), and covered by a dark sheet before and between measurements. A measure of baseline pupil size (for the calculation of change metrics) was taken for 3 seconds preceding the onset of light stimuli for each measurement. In the case of a measurement being compromised due to a loss of the pupil image (typically due to blinking) participants repeated the entire protocol, including the 10-minute dark adaption period.

### Pupillometry

The Neuroptics PLR-2000 (Neuroptics, Irvine, CA, USA) was used to conduct all pupillometry assessments. This device is Therapeutic Goods Administration (TGA) approved, and allows customised measures of the PLR and steady-state pupil size using an infrared camera. The device takes monocular measurements, and all measurements in this study were conducted on the left eye. The in-built light source (14 white LEDs, CCT: 7192 peak nm: 449) was used to deliver two 1-second light stimuli, described here as dim (~10 lux) and bright (~1500 lux). The relative retinal impact for each light stimuli is presented in Table 2, as per Lucas et al [17].

**Table 2:** Effective illuminance for human photopigments, and total irradiance for each light stimulus.

	Irradiance μW/cm^2^	Photopic lux	Cyanopic lux	Melanopic lux	Rhodopic lux	Chloropic lux	Erythropic lux
Dim	4.86	13.00	16.30	12.03	12.36	12.80	12.57
Bright	476.36	1,417.78	1,832.76	1,294.79	1,337.42	1,392.80	1,364.59

### Pupillary light reflex metrics

The two primary outcome metrics were mean constriction velocity (CV) and maximum CV. The mean CV is the average speed of the constriction in mm/s between the onset of constriction and peak constriction for the measurement. Maximum CV represents the fastest 33ms epoch during the constriction period (between onset and max constriction) in mm/s. Both of these metrics are automatically calculated by the device immediately following a successful, artefact free measurement.

### Data analysis

One-way analyses of variance were used to examine differences in constriction velocity between circadian DSPDs, non-circadian DSPDs and healthy controls. Although our sample sizes were not equal, this did not result in heterogeneity of variances between groups. Post-hoc analyses were conducted using a Bonferroni-Holm correction [18]. Receiver Operating Characteristic (ROC) curves were generated to determine the utility of each of our pupil constriction metrics in determining DSPD phenotype and generate potential cut-off scores for clinical use using Sigmaplot v13.0 (Systat Software, San Jose, CA, USA). For *n* = 1 circadian DSPD patient only dim mean CV could be determined; analyses for dim max CV, bright mean CV and bright max CV therefore include *n* = 13 circadian DSPD patients.

## Results

### Between group comparisons

ANOVAs revealed significant between group differences for mean constriction to dim light, and mean and max constriction to bright light. The ANOVA for max constriction to dim light was approaching significance (*p* = .051). Post-hoc analysis for the three significant ANOVAs showed that circadian DSPD patients exhibited faster constriction velocity on all outcomes, compared to non-circadian DSPD patients. Circadian DSPD patients also exhibited a faster PLR than healthy controls for bright light exposures, while non-circadian DSPD patients did not differ from controls on any outcomes (see Table 3). Although bedtime differed between groups, it did not correlate with any pupil constriction outcomes (*r’s* < .18, *p*’s>.11), and so was not considered a possible covariate.

**Table 3:** Results from ANOVAs for constriction velocity and subsequent pairwise comparisons.

	Constriction velocity (mm/s)			
	Dim Mean	Dim Max	Bright Mean	Bright Max
Circadian DSPD	2.62 (0.31)	4.59 (0.43)	2.86 (0.31)	5.19 (0.80)
Non-circadian DSPD	2.21 (0.38)	3.99 (0.61)	2.38 (0.32)	4.25 (0.56)
Healthy controls	2.39 (0.34)	4.33 (0.63)	2.59 (0.38)	4.52 (0.79)
*p* value	.01	.051	.005	.006
Sig pairwise comparisons	CvNC**	N/A	CvNC**, CvH*	CvNC**, CvH*

Error denoted in SD, C = circadian DSPD, NC = non-circadian DSPD, H = healthy controls

* *p* < .05

** *p* < .01, pairwise comparisons conducted using a Bonferroni-Holm correction.

### ROC curves for circadian vs. non-circadian DSPD phenotypes

**Fig 1:**
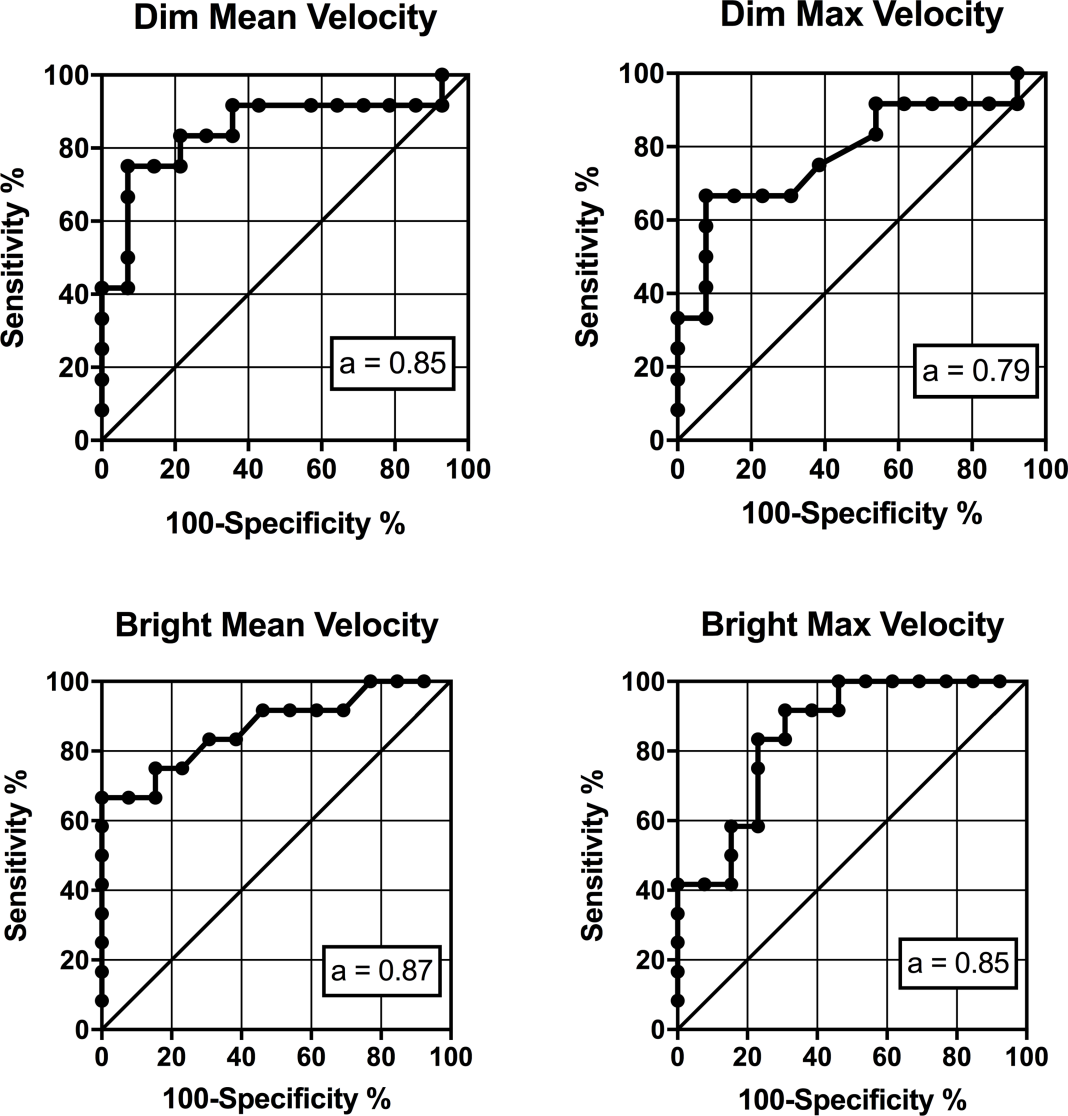
ROC curves demonstrating the sensitivity and specificity of the mean and maximum pupil constriction velocity to dim and bright light in determining circadian vs. non-circadian DSPD. *a* = area under the curve with a value closer to 1 representing better predictive value.

Each of the ROC curves and the associated area under the curve (AUC) values are presented in Fig 1. The optimal cut-off scores (determined using Youden’s Index [19]) for each of these curves, with associated specificity and sensitivities, are presented in Table 4. The highest overall predictive value (as measured by total AUC) was achieved with the mean constriction velocity of the bright light pulse exposure.

**Table 4:** Sensitivity and specificity metrics for each of the Receiver Operating Characteristic curves.

	Sensitivity (%)	Specificity (%)	AUC	Optimal cut-off	*p-*value
Dim Mean CV	92.86	75	0.85 (.09)	2.26	.002
Dim Max CV	92.31	66.67	0.79 (.10)	4.11	.015
Bright Mean CV	100	66.67	0.87 (.08)	2.45	.002
Bright Max CV	69.23	91.67	0.85 (.08)	4.93	.003

Optimal cut-off scores and associated sensitivity/specificity calculated using a pre-test probability of 50%, and a cost-ratio of 1. Error for AUC values denoted as standard error.

## Discussion

This study investigated the utility of the PLR as a method of differentiating DSPD phenotypes, and distinguishing DSPD patients from healthy controls. We were able to successfully predict DSPD phenotype using a simple cut-off score for the mean and maximum constriction velocity of the PLR to both dim and bright light pulses. As hypothesised, circadian DSPD patients consistently demonstrated a faster PLR than non-circadian DSPD patients. Only circadian DSPD patients were distinguished from healthy controls using bright light PLR outcomes, exhibiting a significantly faster PLR than healthy controls, which may indicate a hypersensitivity to light. Non-circadian DSPD patients exhibited a ‘typical’ PLR, which was similar to that of our control participants. These findings suggest that PLR may be a useful clinical test in the diagnosis of DSPD phenotype.

Although our DSPD patients and healthy controls differed in age, this is unlikely to explain the observed differences in the PLR between groups. Previous studies have demonstrated reductions in mean and max constriction velocity in elderly patients [20]. Our groups each fell below a mean age of 30 years, and are unlikely to be demonstrating such age-related changes. Additionally, our circadian DSPD patients exhibited an increase in constriction velocity relative to our younger group of healthy controls, which is counter to any age-related change which could be expected.

The relationship between pupil responses and sleep-wake timing has been attributed to the direct stimulation of melanopsin containing ipRGCs [13, 14]. Previous studies have been designed to distinguish the action of melanopsin from the action of rods and cones [8, 21, 22]. However, our results suggest a rod cone-mediated pathway may be informative in distinguishing individual differences in circadian light sensitivity, and therefore sleep-wake outcomes. The PLR is mediated by melanopsin-containing ipRGCs, which receive additional light input from rods and cones. Optimal activation of ipRGCs occurs with bright, long duration light stimuli [21, 23]. However, ipRGC activation may also occur using short duration light exposures of high light intensity [22]. Our findings suggest that adequate activation of these cells can occur through a rod-cone mediated pathway alone. Our bright, 1-second exposure may have had adequate intensity to activate melanopsin directly [22], however, it is highly unlikely that there was melanopsin activation with our dim light stimulus (~10 lux), which still successfully differentiated the DSPD phenotypes. Thus, the PLR produced by the activation of rods and cones alone using short, dim light pulses alone may provide highly informative clinical data. This is of high clinical interest, as the PLR can be produced with commercially available devices and does not require any particular expertise to calculate or interpret measurement outcomes.

In clinical practice, the misdiagnosis of sleep disorders as a result of common symptomology is a prevalent issue [24]. DSPD patients are often miscategorised as insomnia patients, due to seemingly similar presentations when adequate sleep opportunity at the desired sleep time is not present [1, 3]. A recent study reported that up to 22% of insomnia patients have abnormal circadian timing relative to the sleep-wake cycle, suggesting a circadian mechanism for their sleep disturbances [25]. Further, the two phenotypes of DSPD receive the same clinical diagnosis based on behavioural symptomology, despite exhibiting distinct physiology (i.e., differences in circadian timing). It has been previously reported that DSPD patients exhibit a hypersensitivity to night-time light exposure [6], as measured by melatonin suppression. This hyper-sensitivity to light may result in a circadian system that is more vulnerable to the phase-shifting effects of night-time light exposure, leading to a circadian phase that is abnormally delayed. However, it may be that only patients who present with a circadian delay relative to the desired or required sleep-wake cycle exhibit this abnormal sensitivity to light. It’s likely that this hypersensitivity to light results in the persistently late circadian and sleep phases seen in circadian DSPD patients. In our study, the circadian DSPD patients exhibited a hypersensitivity of the PLR to both dim and bright light, while non-circadian patients exhibited a response that was similar to healthy controls. Therefore, the increased PLR may reflect a difference in the underlying physiology of these two phenotypes, which results in two distinct patterns of circadian timing relative to sleep.

Current guidelines for the treatment of DSPD recommend morning bright light therapy, and evening administration of melatonin [3]. Both of these interventions are designed to achieve an advance in phase, to correct the presumed underlying circadian delay in DSPD patients. As non-circadian DSPD patients lack a circadian delay relative to the desired sleep-wake cycle, phase advancing the circadian pacemaker with bright light or melatonin is unlikely to be efficacious in treating the disorder (although this remains to be tested). Administration of melatonin can have a sleep promoting effect independent of its effects on circadian phase, as demonstrated by a shorter sleep onset latency and increased sleep efficiency [26, 27]. However, when administered at times when endogenous melatonin is present, no improvements in sleep quality are seen [28]. There are no specific guidelines as to *when* melatonin should be administered for the treatment of DSPD. Administration times which range from relative to DLMO (5 hours prior), relative to bedtime (20 minutes prior), to fixed clock times (ranging from 19:35–22:00 h) have been reported [29]. Non-circadian DSPD patients do not exhibit a circadian delay relative to their desired bedtime [2]. Therefore, treatments designed to advance the pacemaker, or administration of melatonin close to bedtime when endogenous levels are high, would be unlikely to produce clinical benefits. Non-circadian DSPD patients more closely resemble the diagnosis of insomnia [1], with sleep-initiation being the primary dysfunction. As such, it may be that these patients benefit more from the recommended insomnia treatment of Cognitive-Behavioural Therapy for Insomnia (CBT-i), with combined short-term hypnotic use in some cases [30]. The non-circadian phenotype of DSPD made up approximately half of all patients in our previous phenotyping study [2], meaning DSPD patients may frequently be receiving interventions which are inappropriate based on the aetiology of their condition.

A potential strength of the PLR in identifying circadian and non-circadian phenotypes within a DSPD patient group is the apparently trait-like nature of our observed abnormality in constriction velocity. This is consistent with the previous finding that the circadian light response is largely genetic [31]. Our categorisation of these patients was based on a phase assessment and clinical diagnosis from ~1.5–2 years prior to their PLR assessment. We found a distinct difference between these two groups, despite an extended period of time elapsing between their clinical diagnosis and DLMO assessment, and the PLR assessment. This suggests that the observed difference in PLR metrics may represent an underlying physiological vulnerability, rather than being reflective of a state-dependent change. The hypersensitivity of the circadian system to light previously observed in DSPD patients [6] may be a trait which makes patients vulnerable to developing the circadian phenotype of DSPD in the presence of late sleep-wake opportunities or excessive night-time light exposure. However, circadian sensitivity to light in individuals with a history of, but not current diagnosis of DSPD, has not been studied. This would aid in better understanding the relationship between circadian light sensitivity, circadian phase and the development of circadian-DSPD.

Although we had up to 87% accuracy in determining DSPD phenotype, not all circadian DSPD patients were captured by our test, and this is likely to be the case in larger samples as well. Some DSPD patients (and patients with other sleep disorders) may exhibit a delay in circadian phase which is not related to a difference in ipRGC function. For example, abnormal SCN activation in response to light, altered behavioural patterns in light exposure, or an abnormally short phase advance region of the phase response curve could all result in the circadian DSPD phenotype in the absence of abnormal pupil function. Therefore, a measure such as ours would serve best to confirm a likelihood that sleep disturbance is related to circadian function, rather than to rule out the role of circadian phase in producing sleep disturbance for an individual patient.

In this study, we used a pupillary measure to better-characterise a clinical sleep disorder diagnosis. Of note, DSPD is associated with abnormally high circadian light sensitivity, and pupil responses are a measure of circadian light input. Although further validation of these pupil responses in relation to direct circadian outcomes are required, this study demonstrates the potential for the PLR to be used diagnostically in circadian medicine. Given the measure is brief and has no associated per-use cost, this could be easily adapted into clinical practice. Improved characterisation of these two distinct phenotypes of DSPD will allow for more targeted treatment recommendations, based on the specific aetiologies. This represents a critical step in the shift toward personalised sleep medicine.

## Supporting information

S1 DSPD PLR Dataset (XLSX)
